# Perceived Health as Related to Income, Socio-economic Status, Lifestyle, and Social Support Factors in a Middle-aged Japanese

**DOI:** 10.2188/jea.15.155

**Published:** 2005-09-27

**Authors:** Naren Wang, Motoki Iwasaki, Tetsuya Otani, Rumiko Hayashi, Hiroko Miyazaki, Liu Xiao, Sasazawa Yosiaki, Shosuke Suzuki, Hiroshi Koyama, Tetsuo Sakamaki

**Affiliations:** 1 Medical Informatics and Decision Sciences, Gunma University School of Medicine.; 2 Epidemiology and Prevention Division, Research Center for Cancer Prevention and Screening, National Cancer Center.; 3 Department of Virology and Preventive Medicine, Gunma University School of Medicine.; 4 NPO International Eco-Health Research Group.; 5 Department of Public Health, Gunma University School of Medicine.

**Keywords:** perceived health, Socioeconomic Factors, Social Support, Social Isolation, Life Style

## Abstract

BACKGROUND: Few studies have examined the association of perceived health with socio-economic status, especially income, and social isolation and support in Japan. The purpose of this study is to clarify the associations among perceived health, lifestyle, and socio-economic status, as well as social isolation and support factors, in middle-aged and elderly Japanese.

METHODS: Subjects were 9,650 participants aged 47-77 years who completed a self-administered questionnaire in 2000 in the second survey of a population-based cohort (the Komo-Ise study). The questionnaire included items on sociodemographic and socio-economic factors, social isolation and support, lifestyle, past history of chronic disease and perceived health. Perceived health was dichotomized into excellent or good health and fair or poor health. A logistic regression analysis was used to determine the odds ratios of socio-economic status, social characteristics and lifestyle in relation to self-reported fair or poor health.

RESULTS: We found that household income, physical activity, sleeping, smoking habit, and BMI had a strong association with self-reported fair or poor health in middle-aged and elderly Japanese men and women. Male subjects tended to report fair or poor health as household income decreased. The results for women differed in that social isolation and low social support had a stronger association for self-reported fair or poor health than low household income.

CONCLUSIONS: The results indicated that perceived health was associated with socio-economic and social characteristics among middle-aged and elderly residents in Japan.

Perceived health is an integrated indicator for the subjective assessment of health. Many previous studies have reported that perceived health was strongly associated with health conditions^1^^-^^3^ and mortality,^4^^-^^7^ which suggests it could be a good surrogate marker for individual health. Therefore, factors associated with perceived health have been examined in many populations, particularly in Western populations.^3^^,^^8^^-^^21^ For example, perceived health was associated with lifestyle factors such as smoking habits,^8^^,^^9^ alcohol drinking,^10^^,^^11^ physical exercise,^12^^,^^13^ overweight,^14^ socio-economic status,^15^^-^^19^ and social network and support.^3^^,^^21^

Socio-economic status represented by occupation, educational background, and income is often used in epidemiologic studies.^15^^-^^19^^,^^22^^-^^26^ Pervious studies have consistently shown a significant association of low socio-economic status with both poor perceived health^15^^-^^19^ and high mortality.^24^^-^^26^ A study showed a stronger association of perceived health with income than occupation and education.^15^ Several studies suggested that low income was significantly associated with an increased risk of all-cause and cause-specific mortality.^24^^,^^26^ Moreover, low income might be a predictive for physical, psychological, and cognitive dysfunction.^24^ Although inequality in income in Japan since the late 1980s has increased at a much faster pace than in other industrialized countries, the association of perceived health with socio- economic status, especially income has not been sufficiently investigated in Japan.^27^

We cross-sectionally analyzed data derived from a follow-up survey of a population-based cohort (the Komo-Ise Study^28^^,^^29^) to investigate the association of various factors, including socio-economic factors such as household income, social factors such as social isolation and support, and lifestyle factors such as smoking, with perceived health in middle-aged and elderly men and women.

## METHODS

### Study population

The subjects were members of the Komo-Ise Study, a longitudinal study to examine the relationship between lifestyle, sociodemographic and psychosocial factors and mortality and morbidity, that had followed a cohort of 11,565 middle-aged and elderly Japanese since 1993. The Komo-Ise Study has been described previously.^28^^,^^29^

In 2000, we conducted a follow-up survey for 10,898 subjects (4,280 in Komochi Village and 6,618 in downtown Isesaki City) who were still living out of 11,565 initial participants. Self-administered questionnaires were distributed through the respective municipal offices to the residents of Komochi Village and those living in downtown Isesaki City. The completed questionnaires were collected in sealed envelopes in accordance with the procedure used in the Alameda County Study in 1999. A total of 9,650 residents (3,937 in Komochi Village and 5,713 in downtown Isesaki City) in both areas responded to the second survey (response rate = 88.5%).

The second survey administered in 2000 was a Japanese version of the Alameda County Study 1999 questionnaire.^30^^,^^31^ This Japanese version was partly modified to correspond to the baseline 1993 questionnaire. The items of the Alameda County Study questionnaire were translated from English into Japanese using translation and back-translation involving a bilingual Japanese speaker and a native English speaker. The questionnaire was composed of items dealing with sociodemographic characteristics, health care, chronic disease, activities of daily living, smoking and alcohol drinking habits, lifestyle factors, social networks, social support, mental health, abuse, women’s health problems, and socio-economic status.

### Study variables

The second survey determined the respondent’s perceived health status by asking, “All in all, would you say that your health is generally excellent, good, fair, or poor?” We assigned a dichotomous variable for perceived health (0 if excellent or good; 1 if fair or poor).

Sociodemographic items consisted of gender, age, and location of residence. In relation to health status, the time of the most recent medical check-up was assessed by asking, “Some people get a check-up once in a while, even though they are feeling well and have not been sick. When was the last time you went to a doctor for this kind of check-up when you were not sick: within the last year, 1 or 2 years ago, and more than 2 years ago?” Responses were coded into dichotomous categories: within the last year; or longer than 1 year (1 or 2 years ago and more than 2 years ago).

Lifestyle items consisted of questions on body size, physical activity, alcohol habits, smoking habits, and sleeping. The body mass index (BMI; weight in kilograms divided by the square of height in meters) as a measure of body size was calculated from self-reported data, and was coded into three categories: less than 18.5, 18.5 to less than 25.0, and 25.0 or more. Physical activity was assessed by asking, “Do you engage in physical exercise - often, sometimes, or never?” Alcohol consumption was assessed by asking, “How often do you drink wine, beer, or liquor? The second survey used an alcohol consumption index (unit: drinks/month), based on multiplying the frequency by the quantity. The frequency was assessed based on the answer: never, less than once a week, once or twice a week, three or four times a week, nearly everyday, and everyday, which were coded as 0, 2.5, 6.5, 15, 24, and 30 days per month, respectively. The usual quantity of alcohol was reported as: never, one to less than two units, two to less than five units, and five units or more, and was coded as: 0, 0.6, 1.8, 4.2, and 7.2 drinks, respectively, for sake and wine (1 unit, 180 mL); 0, 0.7, 2.1, 4.9, and 8.4 drinks, respectively, for beer (1 unit, 500 mL can); and 0, 0.5, 1.5, 3.5, and 6 drinks, respectively, for liquor (1 unit, 1 glass). We multiplied the usual quantity scores by the frequency sores to calculated alcohol consumption per month. Those scoring 0 overall were considered abstainers, 1-45 drinks per month were considered to be moderate drinkers, and those over 45 drinks per month were considered to be heavy drinkers.^4^^,^^32^ Smoking habits were assessed by asking, “Have you smoked any cigarettes? Yes, quit, or have never smoked cigarettes regularly.” Sleeping was measured by sleeping hours, as has been done in previous studies.^33^ We assigned a dichotomous variable for sleeping patterns; 0 if 7-8 hours of sleep were reported; 1 if less than 6 hours of sleep or more than 9 hours of sleep were reported.

Socio-economic status items consisted of educational background, occupation, marital status, household income, and household size, i.e., number of persons living in the house together with the respondent. Educational background was selected from the four initial categories (compulsory education, high school, vocational school or special school and junior college, and college or higher), and then classified into two categories: more than 12 years of schooling, i.e., more than high school; and 12 years of schooling or less, i.e., high school or less. Occupation was assessed by asking whether subjects were currently working to earn their income: yes (employment) or no (unemployment). Marital status was categorized into married, separated (those not living together but legally married), divorced (those legally dissolving a marriage), widowed, and single. Income at the individual level was measured as household income, including earnings from work, benefits, and transfer payments on annual household income before taxation. In the analysis, household income was coded into five categories: less then 3.00 million yen, from 3.00 million yen to 4.99 million yen, from 5.00 million yen to 6.99 million yen, from 7.00 million yen to 9.99 million yen, and 10.00 million yen or more.

Social support items consisted of three questions asking study subjects the availability of some supports as follows: “someone to confide in or talk to about yourself or your problems,” “someone to take you to the doctor,” and “someone to prepare meals for you.” The availability of these items was then classified into five categories: all of the time; most of the time; some of the time; a little of the time; or none of the time. Finally, social support items were classified into three categories by availability: all of the time; most of the time and some of the time; and a little of the time and none of the time. Social isolation was defined as having fewer than three close friends or fewer than three close relatives and seeing fewer than three friends or relatives at least once a month. Participants to whom at least two of these applied were classified as being socially isolated.^32^^,^^34^

### Statistical analysis

We used an unconditional logistic regression model to estimate odds ratios (ORs) and their 95% confidence intervals (CIs) for self-reported fair or poor health to interest factors including lifestyle, socio-economic status, social support and isolation, using SPSS^®^ version 11.5J for Windows. All analyses were performed separately for men and women. We calculated age- and area-adjusted ORs and multivariate ORs. Explanatory variables for these analyses were as follows: age (10-year age categories), area (Komochi Village [rural] and Isesaki City [urban]), educational background, occupation, marital status, household size, household income, physical activity, sleeping, alcohol habit, smoking habit, check-up, BMI, social isolation, and social support items. We used these variables after categorizing as mentioned above in the Study variables section. The test of linear trends was estimated by treating each category as an ordinal variable. P values for the linear trend were evaluated by a two-sided test with 0.05 as a statistically significant level.

## RESULTS

Overall, 55.2% of men reported excellent or good health and 44.8% reported fair or poor health, whereas 51.7% of women reported excellent or good health, and 48.3% of women reported fair or poor health (Table 1).

**Table 1.** Distribution of age, area, and perceived health separated by sex.

	Men		Women	
	
	No.	%	No.	%
Age (year)				
47-49	321	7.1	306	6.0
50-59	1650	36.5	1731	33.8
60-69	1519	33.6	1879	36.6
70-77	1033	22.8	1211	23.6

Area				
Urban	2588	57.2	3125	61.0
Rural	1935	42.8	2002	39.0

Perceived health				
Excellent	565	12.7	501	9.9
Good	1899	42.5	2119	41.8
Fair	1825	40.9	2261	44.6
Poor	175	3.9	184	3.6

We showed the association of the factors of interest with self-reported fair or poor health in men (Table 2) and women (Table 3). Although data from 2,542 men and 2,634 women were analyzed because of the list-wise subject deletion in multivariate analyses in these tables, the results of age- and area-adjusted analyses did not change when limited to same subjects used in these multivariate analyses.

**Table 2.** Age- and area-adjusted and multivariate odds ratios for self-reported fair or poor health in men.

Variable	Fair or poor (%)	Age- and area-adjusted*		Multivariate^†^	
	
		Odds ratio (95% confidence interval)	p for trend	Odds ratio (95% confidence interval)	p for trend
Age (year)					
47-49	133 (41.7)	1.00 reference	0.03	1.00 reference	0.48
50-59	704 (43.1)	1.05 (0.82-1.35)		1.07 (0.74-1.37)	
60-69	645 (42.9)	1.02 (0.79-1.31)		0.85 (0.61-1.19	
70-77	518 (51.4)	1.41 (1.08-1.83)		0.91 (0.62-1.32)	

Area					
Urban	1213 (47.3)	1.00 reference		1.00 reference	
Rural	787 (41.5)	0.80 (0.71-0.91)		0.77 (0.64-0.92)	

Education					
More than high school	394 (46.1)	1.00 reference		1.00 reference	
High school or less	1541 (44.5)	0.97 (0.83-1.13)		0.94 (0.77-1.15)	

Occupation					
Employment	1256 (40.4)	1.00 reference		1.00 reference	
Unemployment	701 (54.9)	1.84 (1.58-2.15)		1.76 (1.42-2.19)	

Household income (million yen/year)					
less than 3.00	385 (51.9)	1.74 (1.40-2.16)		1.53 (1.13-2.07)	
3.00 to 4.99	419 (47.0)	1.41 (1.15-1.73)		1.23 (0.94-1.60)	
5.00 to 6.99	307 (45.4)	1.36 (1.10-1.69)		1.33 (1.02-1.73)	
7.00 to 9.99	302 (42.8)	1.21 (0.97-1.49)		1.13 (0.88-1.47)	
10.00+	277 (38.5)	1.00 reference	<0.0001	reference	0.07

Marital status					
Married	1683 (44.2)	1.00 reference		1.00 reference	
Separated	10 (45.5)	1.06 (0.46-2.48)		0.95 (0.31-2.93)	
Divorced	59 (55.1)	1.61 (1.09-2.38)		0.91 (0.51-1.63)	
Widowed	81 (42.0)	0.82 (0.61-1.11)		0.83 (0.54-1.29)	
Single	119 (51.3)	1.44 (1.10-1.89)		1.02 (0.67-1.56)	

Household size					
per each additional person	1929 (44.8)	0.95 (0.91-0.99)		1.03 (0.97-1.09)	

Physical activity					
Often	234 (34.6)	1.00 reference	<0.0001	1.00 reference	<0.0001
Sometimes	493 (44.1)	1.56 (1.28-1.91)		1.67 (1.29-2.14)	
Never	1114 (49.2)	1.94 (1.62-2.33)		1.85 (1.46-2.33)	

Sleeping					
7 or 8 hrs	1407 (42.7)	1.00 reference		1.00 reference	
less than 6 or 9+ hrs	573 (50.8)	1.38 (1.20-1.58)		1.36 (1.13-1.64)	

Alcohol habit					
Abstain	575 (51.7)	1.00 reference	<0.0001	1.00 reference	0.006
Moderate (1-45 drinks per month)	559 (46.9)	0.84 (0.71-0.99)		0.91 (0.74-1.12)	
Heavy (over 45 drinks per month)	484 (39.1)	0.63 (0.53-0.74)		0.72 (0.59-0.89)	

Smoking habit					
Current	909 (44.1)	1.10 (0.93-1.31)		0.94 0.75-1.18)	
Former	712 (47.0)	1.18 (0.99-1.41)		1.08 0.85-1.36)	
Never	325 (41.7)	1.00 reference	0.18	1.00 reference	0.36

Check-up					
Within the last year	1693 (45.1)	1.00 reference		1.00 reference	
Not with the last year	257 (44.4)	0.96 (0.81-1.15)		0.93 (0.73-1.20)	

Body mass index (kg/m^2^)					
less than 18.5	120 (60.0)	1.84 (1.37-2.48)		1.77 (1.18-2.65)	
18.5-24.9	1338 (43.0)	1.00 reference		1.00 reference	
25.0+	478 (47.0)	1.19 (1.04-1.38)		1.18 (0.97-1.43)	

Social isolation					
No isolation	1479 (43.1)	1.00 reference		1.00 reference	
Isolation	400 (52.5)	1.46 (1.24-1.71)		1.12 (0.90-1.40)	

Someone to confide in or talk to about yourself or your problems					
All of the time	901 (42.5)	1.00 reference	0.001	1.00 reference	0.61
Most of the time & some of the time	679 (46.0)	1.77 (1.03-1.35)		1.11 (0.91-1.35)	
A little of the time & none of the time	342 (50.4)	1.38 (1.16-1.64)		1.05 (0.80-1.37)	

Someone to take you to the doctor					
All of the time	1266 (42.7)	1.00 reference	<0.0001	1.00 reference	0.36
Most of the time & some of the time	523 (48.7)	1.29 (1.12-1.48)		1.13 (0.90-1.44)	
A little of the time & none of the time	162 (55.5)	1.67 (1.31-2.14)		1.29 (0.85-1.94)	

Someone to prepare meals for you					
All of the time	1383 (43.6)	1.00 reference	0.0001	1.00 reference	0.27
Most of the time & some of the time	401 (46.6)	1.16 (0.99-1.35)		1.12 (0.88-1.43)	
A little of the time & none of the time	158 (56.0)	1.65 (1.29-2.11)		1.38 (0.91-2.10)	

* : Adjusted for age (47-49, 50-59, 60-69, 70-77) and area (urban and rural)

† : Adjusted for age, area, education, occupation, marital status, household, household income, physical activity, sleeping, alcohol habit, smoking habit, check-up, BMI, social isolation and social support factors.

**Table 3.** Age- and area-adjusted and multivariate odds ratios for self-reported fair or poor health in women.

Variable	Fair or poor (%)	Age- and area-adjusted*		Multivariate^†^		
	
		Odds ratio (95% confidence interval)	p for trend	Odds ratio (95% confidence interval)	p for trend
Age (year)					
47-49	133 (43.5)	1.00 reference	<0.0001	1.00 reference	0.21
50-59	776 (45.3)	1.08 (0.85-1.39)		1.09 (0.80-1.50)	
60-69	875 (47.0)	1.12 (0.88-1.44)		0.99 (0.71-1.40)	
70-77	661 (55.8)	1.60 (1.23-2.07)		1.45 (0.99-2.13)	

Area					
Urban	1547 (49.9)	1.00 reference		1.00 reference	
Rural	898 (45.7)	0.85 (0.75-0.95)		0.83 (0.69-0.99)	

Education					
More than high school	339 (45.4)	1.00 reference		1.00 reference	
High school or less	2006 (49.0)	1.12 (0.95-1.31)		1.14 (0.93-1.41)	

Occupation					
Employment	920 (42.1)	1.00 reference		1.00 reference	
Unemployment	1430 (53.4)	1.49 (1.31-1.70)		1.55 (1.30-1.86)	

Household income (million yen/year)					
less than 3.00	508 (52.6)	1.56 (1.27-1.93)		1.22 (0.92-1.63)	
3.00 to 4.99	428 (49.8)	1.45 (1.17-1.79)		1.25 (0.96-1.63)	
5.00 to 6.99	293 (50.7)	1.53 (1.22-1.92)		1.39 (1.05-1.82)	
7.00 to 9.99	289 (45.5)	1.27 (1.01-1.58)		1.09 (0.84-1.42)	
10.00+	260 (39.6)	1.00 reference	<0.0001	1.00 reference	0.18

Marital status					
Married	1744 (47.0)	1.00 reference		1.00 reference	
Separated	15 (51.7)	1.20 (0.57-2.49)		0.79 (0.29-2.11)	
Divorced	87 (53.7)	1.28 (0.93-1.75)		0.93 (0.60-1.45)	
Widowed	403 (52.6)	1.09 (0.93-1.29)		1.02 (0.80-1.31)	
Single	120 (51.7)	1.14 (0.87-1.49)		1.06 (0.71-1.58)	

Household size					
per each additional person	2370 (48.2)	0.96 (0.92-0.99)		1.02 (0.96-1.08)	

Physical activity					
Often	254 (39.3)	1.00 reference	<0.0001	1.00 reference	<0.0001
Sometimes	610 (45.6)	1.33 (1.10-1.62)		1.17 (0.90-1.51)	
Never	1307 (52.5)	1.79 (1.50-2.14)		1.59 (1.25-2.03)	

Sleeping					
7 or 8 hrs	1555 (44.9)	1.00 reference		1.00 reference	
less than 6 or 9+ hrs	856 (55.9)	1.57 (1.39-1.77)		1.5 (1.26-1.78)	

Alcohol habit					
Abstain	1550 (50.8)	1.00 reference	0.02	1.00 reference	0.09
Moderate (1-45 drinks per month)	568 (45.6)	0.84 (0.74-0.96)		0.87 (0.73-1.04)	
Heavy (over 45 drinks per month)	42 (35.9)	0.58 (0.40-0.86)		0.67 (0.43-1.04)	

Smoking habit					
Current	256 (51.2)	1.20 (1.00-1.45)		1.14 (0.86-1.51)	
Former	123 (58.0)	1.51 (1.14-2.00)		1.70 (1.11-2.60)	
Never	1751 (47.0)	1.00 reference	0.004	1.00 reference	0.04

Check-up					
Within the last year	2055 (47.8)	1.00 reference		1.00 reference	
Not with the last year	290 (52.7)	1.22 (1.02-1.46)		1.19 (0.91-1.55)	

Body mass index (kg/m^2^)					
less than 18.5	178 (61.0)	1.80 (1.40-2.30)		1.57 (1.11-2.23)	
18.5-24.9	1556 (45.1)	1.00 reference		1.00 reference	
25.0+	637 (54.2)	1.44 (1.26-1.65)		1.34 (1.10-1.62)	

Social isolation					
No isolation	1902 (46.7)	1.00 reference		1.00 reference	
Isolation	337 (59.5)	1.68 (1.41-2.02)		1.60 (1.22-2.10)	

Someone to confide in or talk to about yourself or your problems					
All of the time	1428 (45.2)	1.00 reference	<0.0001	1.00 reference	0.63
Most of the time & some of the time	717 (53.3)	1.41 (1.24-1.61)		1.09 (0.90-1.33)	
A little of the time & none of the time	198 (58.1)	1.65 (1.32-2.07)		1.12 (0.78-1.63)	

Someone to take you to the doctor					
All of the time	1578 (44.8)	1.00 reference	<0.0001	1.00 reference	0.01
Most of the time & some of the time	687 (56.8)	1.67 (1.46-1.91)		1.39 (1.10-1.76)	
A little of the time & none of the time	115 (61.5)	1.93 (1.42-2.62)		1.59 (0.93-2.71)	

Someone to prepare meals for you					
All of the time	1310 (43.9)	1.00 reference	<0.0001	1.00 reference	0.07
Most of the time & some of the time	845 (54.6)	1.58 (1.39-1.79)		1.25 (1.01-1.55)	
A little of the time & none of the time	215 (57.8)	1.72 (1.38-2.14)		0.93 (0.63-1.36)	

* : Adjusted for age (47-49, 50-59, 60-69, 70-77) and area (urban and rural)

† : Adjusted for age, area, education, occupation, marital status, household, household income, physical activity, sleeping, alcohol habit, smoking habit, check-up, BMI, social isolation and social support factors.

### Sociodemographic and Socio-economic Factors

With respect to socio-economic status variables, no association was observed between educational background and self-reported fair or poor health but unemployment was significantly associated with self-reported fair or poor health in the multivariate model for both men and women (Tables 2 and 3). In men, the multivariate OR for reporting fair or poor health was 1.53 (95% CI: 1.13-2.07) for the lowest household income category (<3.00 million yen; Table 2), compared to the highest household income category (10.00+ million yen). The linear trend was borderline significant (P for trend = 0.07). Although age- and area-adjusted OR for reporting fair or poor health was 1.56 (95%: CI 1.27-1.93) in the lowest household income category (<3.00 million yen; P for trend < 0.0001), multivariate ORs were not statistically significant in women (Table 3). Marital status and household size were not significantly associated with self-reported fair or poor health for either men or women.

### Lifestyle Factors

In terms of lifestyle variables, physical activity (sometimes and never) in both men and women, sleeping (<6 or 9+ hrs) in both men and women, low BMI (<18.5) and high BMI (25.0+) in both men and women, and smoking habit (current and former) in women were significantly associated with self-reported fair or poor health in the age- and area-adjusted model (Tables 2 and 3). However, no statistical significance was found for physical activity (sometimes) in women, high BMI (25.0+) in men, and smoking habit (current) in women after adjustment. On the other hand, a heavy drinking habit was significantly related to decreased ORs for self-reported fair or poor health in both men and women, but not for women in the multivariate model. Neither smoking in men nor medical check-up (within the last year) in both men and women were not significantly associated with self-reported fair or poor health.

### Social Isolation and Support Factors

With respect to social isolation and support variables, social isolation was significantly associated with self-reported fair or poor health in women, while in men social isolation was significantly associated with self-reported fair or poor health in the age- and area-adjusted model but not in the multivariate model. Three social support items were significantly associated with self-reported fair or poor health in men in the age- and area-adjusted model but not in the multivariate model. On the contrary, one social support item, having someone to take him/her to physicians, was associated with self-reported fair or poor health in women even in the multivariate model.

### Stratified Analysis by Study Area

We performed a stratified analysis by study area. In urban men, low household income was significantly associated with self-reported fair or poor health. On the contrary, there was no significant association between low household income and self-reported fair or poor health in rural men. The association between household income and self-reported fair or poor health did not differ between urban and rural women (Table 4). Factors other than household income did not differ in relation to self-reported fair or poor health between urban and rural area.

**Table 4.** Multivariate odds ratios for self-reported fair or poor health separeted by study area.

Household income (million yen/year)	Urban		Rural	
	
	Odds ratio^†^ (95% confidence interval)	p for trend	Odds ratio^†^ (95% confidence interval)	p for trend
	Men			
less than 3.00	2.22 (1.50-3.28)		0.79 (0.47-1.35)	
3.00 to 4.99	1.20 (0.86-1.68)		1.28 (0.82-2.01)	
5.00 to 6.99	1.28 (0.91-1.80)		1.37 (0.89-2.13)	
7.00 to 9.99	1.09 (0.79-1.51)		1.21 (0.78-1.87)	
10.00+	1.00 reference	0.001	1.00 reference	0.15
	Women			
less than 3.00	1.36 (0.96-1.94)		1.02 (0.62-1.69)	
3.00 to 4.99	1.33 (0.96-1.84)		1.16 (0.72-1.86)	
5.00 to 6.99	1.35 (0.95-1.91)		1.43 (0.90-2.28)	
7.00 to 9.99	1.15 (0.83-1.59)		0.99 (0.63-1.56)	
10.00+	1.00 reference	0.37	1.00 reference	0.46

† : Adjusted for age, area, education, occupation, marital status, household, household income, physical activity, sleeping, alcohol habit, smoking habit, check-up, BMI, social isolation and social support factors.

## DISCUSSION

Our results showed that household income in men, social isolation and support in women, and some lifestyle factors in both men and women, such as physical activity, sleep, smoking, and BMI, had a strong association with self-reported fair or poor health in middle-aged and elderly Japanese. Male subjects tended to report fair or poor health, as household income decreased. Social isolation and low social support had a stronger association for self-reported fair or poor health than low household income in women, but not in men. These results did not substantially change after excluding subjects with a history of heart disease, cerebrovascular disease, or cancer (data not shown).

This study showed the significant association of low household income with self-reported fair or poor health in men, although no association was found for education. Shibuya, et al.^18^ reported that individual income had a stronger association with self-rated health than income inequality at the prefecture level in Japan. Lynch, et al.^24^ found that, in the long term, sustained low income has an impact on the onset of severe health problems. The result of our study was consistent with the findings from previous studies.^15^^,^^18^^,^^19^ However, the stratified analysis suggested a significant association of low household income with self-reported fair or poor health in urban men but not in rural men. This might be a chance finding due to the smaller number of subjects after the stratification. Otherwise, household income might be a more important factor related to perceived health in urban men than rural men.

We observed a different association of household income with self-reported fair or poor health between men and women, although some previous studies showed no gender difference.^15^^,^^19^ In contrast to the finding in men, in women the association of household income was attenuated by adjustment for social isolation and social support factors. These social factors may have a stronger association for self-reported fair or poor health than low household income in women. The household income may have a more important role in relation to self-reported fair or poor health in men than women, because the association persisted after adjustment for other confounding factors in men.

Unemployment was significantly associated with self-reported fair or poor health in both men and women in our study, independently of household income, education, social isolation and support, and lifestyle factors. The proportion of unemployment in women was very different from men. However, the effect of unemployment on perceived health did not differ between men and women, which was consistent with previous reports.^15^^,^^19^

In this study, subjects were less likely to report their fair or poor health as alcohol consumption or physical activity increased, which is consistent with previous studies.^10^^-^^13^ Although being overweight was associated with self-reported fair or poor health in Western populations,^14^^,^^21^ we found that the lean group (BMI<18.5) had a significant association with self-reported fair or poor health. Lee, et al.^35^ reported that overweight men had an elevated risk of all-cause mortality and no evidence of excess risk among lean men. However, Tsugane, et al.^36^ showed an increased risk of all-cause mortality for not only overweight but lean Japanese. This finding implies that being underweight may also contribute to poor health.

Some reports have linked social networks or social support with mortality.^37^^,^^38^^,^^39^^,^^40^ Social networks are an important predictor of mortality risk for middle-aged and elderly Japanese men and women.^37^ Welin, et al.^38^^,^^39^ reported that high levels of social, home, and outside home activities protected middle-aged men from premature death. Reynolds, et al.^40^, reported that socially isolated women were at a significantly elevated risk of cancer mortality. Our study showed, in women only, that social isolation or a social support item that having someone to take her to the doctor was significantly associated with self-reported fair or poor health after controlling for other factors including household income. This finding is thus in accord with those previous studies.

There are several limitations to the present study. First, household income may be a very sensitive question for Japanese respondents, because the response rate for the question on household income was 77.3%, compared with the overall questionnaire response rate of 88.5%. Since many of the residents who did not provide the answer for household income could well have been in one of the lower income groups, this could have affected the results. Second, since this was a cross-sectional study, it is difficult to draw conclusions about any causal relationships that may exist between perceived health and the various factors. In the future, it will be necessary to accumulate evidence from prospective studies to clarify the causal relationships between perceived health and the various factors.

Overall, however, this study provided evidence that socio-economic and social characteristics were important factors that relate to the perceived health of middle-aged and elderly residents in Japan.
